# Functional characterization of a biallelic *MIPEP* variant associated with global developmental delay, infantile epileptic spasms syndrome, and hypotonia

**DOI:** 10.1016/j.ymgmr.2026.101335

**Published:** 2026-06-24

**Authors:** Benedetta Ruzzenente, Pierre-Hadrien Becker, Elissa Afram, Pauline Gaignard, Agnes Rötig, Denis M. Nyaga, Cynthia Sharpe, Lynette G. Sadleir, Bryony Ryder, Metodi D. Metodiev

**Affiliations:** a Genetics of rare ophthalmological, auditory, and mitochondrial disorders, INSERM UMR1163, Université Paris Cité, Institut Imagine, Paris, France; b Service de Biochimie, UF Maladies Héréditaires du Métabolisme, CHU Bicêtre, AP-HP, Le Kremlin-Bicêtre, Paris, France; c Liggins Institute, The University of Auckland, Auckland, New Zealand; d Department of Neuroservices, Te Toka Tumai, Te Whatu Ora, Auckland, New Zealand; e Department of Paediatrics, University of Otago, Wellington, New Zealand; f Paediatric and Adult National Metabolic Service, Te Toka Tumai, Te Whatu Ora, Health New Zealand, Auckland, New Zealand

**Keywords:** Mitochondrial intermediate peptidase, *MIPEP*, MIP, Mitochondrial disease, OXPHOS assembly defect, Mitochondrial proteostasis, COXPD31

## Abstract

The mitochondrial intermediate peptidase (MIP) catalyzes the post-import removal of an N-terminal octapeptide from a subset of nuclear-encoded mitochondrial proteins. While the mechanistic role of this processing remains unclear, biallelic *MIPEP* variants have been linked to respiratory chain dysfunction and mitochondrial disease. Patients expressing these variants most often presented with cardiomyopathy, variable neurological defects, and early mortality. Here, we report the identification and functional characterization of a homozygous *MIPEP* variant in a patient presenting with a comparatively milder clinical phenotype involving global developmental delay, infantile epileptic spasms syndrome, and hypotonia. Analyses of patient-derived fibroblasts revealed reduced MIP abundance and impaired processing of established MIP substrates MRPL12, NDUFV2, and ATP5F1. Expression of wild-type *MIPEP* restored these defects, confirming the pathogenic nature of the variant. Thus, our findings expand the genetic and phenotypic spectrum of *MIPEP*-linked disease.

## 1 Introduction

Most mitochondrial proteins are encoded by nuclear genes, synthesized in the cytosol, and imported into mitochondria, often via N-terminal mitochondrial leader peptides. The matrix processing peptidase (MPP) removes the leader peptide during import, which is usually sufficient to generate a mature and functional protein. However, certain newly imported proteins require a second proteolytic step such as the removal of 8 amino acids by the mitochondrial intermediate peptidase (MIP, encoded by *MIPEP*) or of a single amino acid by the X-prolyl aminopeptidase (XPNPEP3). We and others have shown that MIP-mediated processing plays a critical role in the biogenesis of the oxidative phosphorylation system (OXPHOS) although the exact mechanism underlying this is unclear.

Inhibition of MIP results in multiple OXPHOS deficiency and mitochondrial disease (combined oxidative phosphorylation deficiency-31 (COXPD31) (OMIM 617228)). To date, just 6 patients with 8 disease-causing variants in the *MIPEP* gene (OMIM 602241) have been reported in the literature [1], [6], [12], [18]. Most patients expressing these variants presented with left ventricular noncompaction cardiomyopathy, failure to thrive and neurological phenotypes, including global developmental delay, seizures, hypotonia and microcephaly (Table 1). Other features include cataract and movement disorder. The presence of cardiomyopathy was associated with increased mortality, with 4 out of 6 patients dying in the first 2 years of life. At the molecular level, studies of the effects of these variants are relatively limited. We have shown that inactivation of MIP led to decreased substrate processing and impaired OXPHOS biogenesis in patient fibroblasts and hypomorphic cell lines [12]. Similarly, others have demonstrated that expression of mutant *Oct1* (the ortholog of *MIPEP*) mimicking known disease-causing *MIPEP* variants in yeast abrogated the processing of several Oct1 substrates [6].

**Table 1:** Summary of the clinical presentations of patients carrying disease-causing variants in *MIPEP*.

Reference, Patient #	Nucleotide change (Protein change)	Cardiomyopathy	Neurology	Other	Biochemistry	Histology	Functional studies	Outcome
**Present article**	**c.890G>C** **p.(G297A)**	**Not present**	**Drug-responsive epilepsy, hypsarrhythmia, CVI, mild inferior vermian hypoplasia, cerebral and cerebellar atrophy, hypotonia**	**DD**	**Fb: normal RC activity and assembly**	**Not performed**	**Fb: impaired substrate processing**	**Alive at 12** **yrs**
Eldomery et al. [6] Patient 1	c.1745 T>G p.(L582R) / c.212 T>A p.(L71Q)	WPW, LVNC	Hypotonia, dystonia, microcephaly, and prominent extra-axial CSF spaces.	DD, FTT, vomiting, constipation	MA, elevated Ala normal RC activity	Muscle: Mt-proliferation, lipid droplets	Yeast: L83Q (MIP L71Q) destabilizes Oct1 mimics KO phenotype	Alive at 4.5 yrs
Eldomery et al. [6] Patient 2	c.916C>T p.(L306F) / c.1804G>T p.(E602*)	LVNC, DCM,	Hypotonia, epilepsy (onset at 1 year)	cataract DD	Mild ↓ CIV activity	Mild fiber size variation, glycogen and lipid accumulation;	Yeast: L339F (MIP L306F) impaired substrate processing	Died at 2 yrs
Eldomery et al. [6] Patient 3	c.1027 A>G p.(K343E)	LVNC, atrial septal defect	Microcephaly, hypotonia, epilepsy (onset 10 months), MRI: bilateral symmetric hyperintensity of the basal ganglia and periventricular white matter.	FTT, MA,	Increased ALT and AST Fb: mild ↓ CI and CIV activity.	Not reported	Yeast: K376E (MIP K343E) impaired substrate processing	Died at 11 mo
Eldomery et al. [6] Patient 4	c.1534C>G p.(H512D) / 1.4 Mb deletion	severe biventricular HCM, pericardial edema,	Neonatal hypotonia, epilepsy (onset on day 1 of life)	DD, congenital abnormalities,	LA, ketosis, hyperinsulinemia Elevations in Ala, Gln, Pro	Muscle: large mitochondrial aggregates, lipid and glycogen deposition	Not reported	Died at 19 days
Pulman et al. [12]	c.916C>T p.(L306F) / c.1970 + 2 T > A p.(A658Lfs*38)	Not present (4, 9 and 14 years)	Hypotonia, mild ataxia, mild optic neuropathy, peripheral neuropathy; mild vermis hypoplasia (3 yrs), bilateral putaminal atrophy (13 yrs), nystagmus	DD, mild facial hypomotility, muscle neck weakness	Fb: ↓ activity of CI; ↓ abundance of CI, CIV and CV	Not performed	Fb and HEK cells: impaired substrate processing	Alive at 20 yrs
Wang et al. [18]	c.1081 T>A p.(Y361N) / Ex1–19 del	HCM	Severe hypotonia, bilateral thalamic and dorsal brainstem abnormalities	DD, genital anomalies,	LA, elevated BNP, ALT, AST ↓ mtDNA in whole blood	Not reported	Not reported	Died at 2 mo

Abbreviations: WPW, Wolf-Parkinson-Syndrome; LVNC, left ventricular noncompaction; DCM, dilated cardiomyopathy; HCM, hypertrophic cardiomyopathy; CVI, cortical visual impairment; FTT, failure to thrive; DD, developmental delay, M/LA, metabolic/lactic acidosis; ALT, alanine transaminase; AST, aspartate transaminase; BNP, plasma B-type natriuretic peptide; RC, respiratory chain; ↓, reduced.

Here, we report the functional characterization of the homozygous c.890G > C variant (ClinVar ID 1806091), which was associated with global developmental delay, infantile epileptic spasm syndrome and hypotonia. Our molecular analyses revealed the accumulation of the intermediate isoforms of previously reported MIP substrates, indicative of an inhibition of MIP-mediated processing. This was in stark contrast to biochemical studies, which revealed normal respiratory chain activities and complex assembly in patient fibroblasts.

## 2 Materials and methods

### 2.1 Patient

**Fig. 1:**
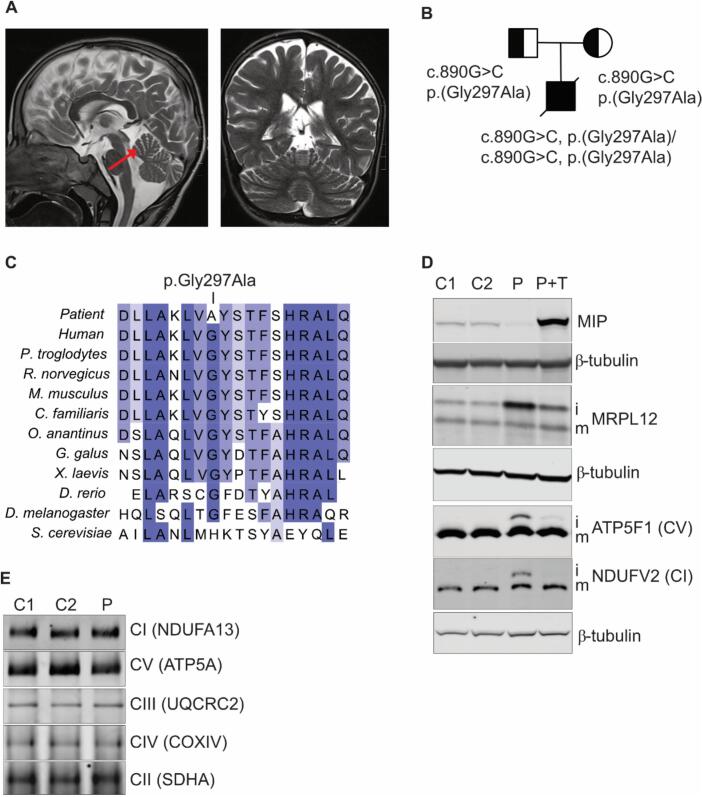
Impaired MIP-mediated processing, but unchanged OXPHOS abundance in patient fibroblasts. A. T2-weighted MRI showing increased prominence of cerebellar folia (red arrow), indicative of volume loss. B. Segregation of the c.890G > C variant in the patient's family. C. Alignment of the MIP polypeptide region containing the identified variant. D. Immunoblotting of protein extracts from control (C) and patient (P) fibroblasts, either mock transduced or complemented with wild-type MIP cDNA (+T). β-tubulin was used as a loading control. i, intermediate isoform; m, mature isoform. E. BN-PAGE analysis of mitoplast extracts from control and patient fibroblasts. Antisera against individual proteins from the five OXPHOS complexes (in brackets) were used. CI-CV, OXPHOS complexes I to V.

The patient, now aged 12 years, is the only child of healthy, non-consanguineous parents of Indian origin. The pregnancy and delivery at term were uncomplicated, birth weight was 3095 g, no resuscitation was required, and there was no history of hypoglycaemia. Family history was non-contributory. Global developmental delay was observed before 3 months of age, and neuroregression at 10 months. At 11 months, he developed epileptic spasms, and when assessed, he was noted to be hypotonic and to have global developmental delay with skills consistent with a 4-month-old. His weight was on the 9th centile, length on 98th centile, head circumference on the 2-9th centile. Brain MRI was normal, and EEG showed hypsarrhythmia. Infantile epileptic spasms syndrome was diagnosed. Prednisone was not effective, vigabatrin was trialled briefly but discontinued due to side effects, while clobazam resulted in epileptic spasm freedom at 16 months and normalisation of his EEG. He had a tonic-clonic seizure during a febrile illness at 20 months but has been seizure free since, despite clobazam being ceased by 3 years of age. Blood lactate was at the upper limit of the normal range (2.2 mmol/L), but normal in cerebrospinal fluid, and there was an initial elevated plasma alanine of 590 umol/L (normal range 175–325 umol/L). Subsequent plasma amino acid levels were normal, and an extensive metabolic workup was non-contributory. Repeat brain MRI at 4 years of age revealed mild inferior vermian hypoplasia and cerebral and cerebellar atrophy (Fig. 1A). An empirical trial with coenzyme Q10 (10 mg/kg/day) was commenced at the request of the patient's parents, despite limited evidence supporting efficacy in this context. At the age of 12 years, he remains seizure-free with slow developmental progress. He is hypotonic but has normal power and mobilises independently without ataxia, having first walked at 7 years of age. He is non-verbal and not toilet-trained, but understands simple instructions. Height and weight have followed the 75th percentile on a pureed oral diet supplemented by PediaSure nutritional supplement. Head circumference is on the 9th centile. He has cortical visual impairment but normal anterior and posterior segments. Serial echocardiograms have ruled out hypertrophic or non-compaction cardiomyopathy. At the age of 5 years, he had a severely dilated aortic root with a moderately dilated aortic annulus and ST junction; however, this has normalized over time, and biventricular function has remained normal.

### 2.2 Exome and whole genome sequencing, and genetic analyses

Clinical exome sequencing was performed by Medgenome (Karnataka, India). Genomic DNA was extracted from peripheral blood samples using standard protocols. Targeted gene capture was performed using Agilent's SureSelect V5 All Exome Capture kit. The resulting libraries were sequenced on an Illumina platform to achieve a mean coverage of 80-100×. Raw sequence reads were aligned to the human reference genome (GRCh37/hg19) using the Burrows-Wheeler Aligner (BWA) [8]. Variant calling was performed using Picard tools and the Genome Analysis Toolkit (GATK) version 3.6, following GATK best practices guideline [17]. Single nucleotide variants (SNVs) and insertions/deletions (indels) were annotated against Ensembl (release 87) using the Variant Effect Predictor (VEP) [9]. Clinically relevant variants were further interpreted with reference to published literature and multiple disease databases, including ClinVar, OMIM, GWAS, HGMD, SwissVar. Common population variants were filtered out based on allele frequencies in 1000 Genomes Phase 3, Exac, EVS, dbSNP147, the 1000 Japanese Genomes, and an internal Indian population database (MAF ≥ 1%). Variant prioritization focused on protein-altering variants, including missense, nonsense, frameshift, and canonical splice-site variants. Synonymous, non-coding, and deep intronic variants were not prioritized unless they were predicted to affect splicing. Pathogenicity predictions were derived using in silico tools, including PolyPhen-2, SIFT, MutationTaster2, Mutation Assessor, and the Likelihood Ratio Test (LRT).

Whole-genome sequencing (WGS) trio analysis was undertaken using genomic DNA extracted from whole blood samples from child and parents. Sequencing was carried out on the Illumina NovaSeq PE150 platform (Novogene, Singapore). Reads were processed as above and aligned to the human reference genome (GRCh38), followed by duplicate marking and base quality score recalibration using GATK (v4.2.4.1). Variant calling was performed with GATK HaplotypeCaller and the identified germline variants annotated using VEP. Variants were retained if their allele frequencies in population databases (i.e., ExAC, gnomAD, and the 1000 Genomes Project) were < 0.0001 for dominant conditions and < 0.01 for recessive conditions. Analysis focused on variants predicted by VEP [9] to have “HIGH” or “MODERATE” functional impact, particularly in epilepsy-associated genes [11]. Potential pathogenicity was further assessed using Varsome in accordance with American College of Medical Genetics and Genomics (ACMG) guidelines [13].

PCR amplification and Sanger sequencing to confirm the variant in fibroblast DNA was performed at the Victorian Clinical Genetics Service, The Royal Children's Hospital, Parkville, Victoria, Australia for variants classified using Agilent Alissa Interpret.

### 2.3 Cell culture

Patient and control dermal fibroblasts were grown at 37 °C in a humidified atmosphere with 5% CO_2_ and were collected at confluence by trypsinization. Cell pellets were frozen in liquid nitrogen and stored at −80 °C until needed.

### 2.4 SDS-PAGE, blue-native-PAGE, and immunoblotting

For SDS-PAGE, proteins were isolated from frozen cell pellets by extraction in RIPA buffer (Thermo Fischer Scientific) as described in [14]. 20–50 μg proteins were fractionated through polyacrylamide gels with different formulations depending on the protein target [12]: 10% or 12% Criterion gels (BioRad) were used for resolving MIP and MRPL12, respectively; high-resolution Tricine-Urea gels were used to resolve the intermediate isoforms of NDUFV2 and ATP5F1. BN-PAGE was performed using 10 μg of control and patient mitoplast extracts in 2% n-dodecyl β-D-maltoside (DDM, Merck) as described in [14]. Immunoblots were carried out using commercially available antisera against: MRPL12 (Proteintech, 14795–1-AP), COXIV (Proteintech, 11242–1-AP), β-tubulin (Proteintech, 10094–1-AP), ATP5F1 (Abcam, ab117991), NDUFV2 (Abcam, ab183715), NDUFA13 (Abcam, ab110240), ATP5A (Abcam, ab14748), UQCRC2 (Abcam, ab14745), SDHA (Abcam, ab14715) and MIP (Abnova, H00004285-M01).

### 2.5 Spectrophotometric analysis of respiratory chain activities

Enzymatic activities were measured by spectrophotometry in permeabilized cells as described in [4].

### 2.6 Functional complementation

Functional complementation was performed using a previously described lentiviral vector, which was stably transduced in patient fibroblasts [12].

## 3 Results

### 3.1 Identification of disease-causing *MIPEP* variants by exome sequencing

Clinical whole exome sequencing identified a homozygous missense variant in exon 7 of *MIPEP* (NM_001330749.2:c.890G > C; p.(Gly297Ala); ENST00000382172). The variant was identified as heterozygous in both unaffected parents (Fig. 1B). This variant results in a glycine-to-alanine substitution at codon 297 within the peptidase family M3 domain of the protein. The c.890G > C variant is absent from the 1000 Genomes and ExAC databases and conserved across species (Fig. 1C). In silico predictions of the variant classified it as probably damaging (PolyPhen-2, HumDiv) and damaging (SIFT, LRT, MutationTaster2). We previously submitted this variant to ClinVar under ClinVar ID 1806091.

Sanger sequencing of fibroblast DNA confirmed the homozygous *MIPEP* variant (NM_005932.4: c.890G > C (p.Gly297Ala)). It was also identified by whole-genome sequencing (Epilepsy Research Group, University of Otago, Wellington). The variant was classified as likely pathogenic using ACMG guidelines [13]. No other candidate genes were identified.

### 3.2 Decreased abundance of mutant MIP and impaired precursor processing in patient fibroblasts

To elucidate the pathogenic potential of the identified *MIPEP* variant, we carried out immunoblot analyses of patient and control fibroblast extracts. These analyses revealed that the abundance of the mutant MIP protein was moderately decreased in patient cells (Fig. 1D). This was accompanied by an accumulation of the intermediate isoforms of several model substrates of MIP: MRPL12, a mitoribosomal protein, and a MIP-deficiency maker [10], [12], NDUFV2 and ATP5F1 subunits of OXPHOS complexes I and V [12], respectively. Expression of the wild-type *MIPEP* cDNA restored the processing of these substrates, reducing the abundance of their intermediate isoform in patient cells (Fig. 1D). These results suggest that the c.890G > C variant decreases MIP protein abundance and impairs MIP-mediated substrate processing in patient cells.

Our previous studies have shown that MIP inhibition resulted in structural instability and decreased steady-state abundance of OXPHOS complexes I, IV, and V in BN-PAGE analyses, but only mild to borderline enzymatic activity defects in complexes I and V in patient fibroblasts [12]. Therefore, we analyzed the assembly of the five OXPHOS complexes by BN-PAGE (Fig. 1E) and measured the activities of respiratory complexes I-IV (Table 2). These analyses revealed that neither the assembly nor activity of the different OXPHOS complexes was affected in the patient's fibroblasts. Similarly, the activities of malate dehydrogenase (MDH, a MIP substrate) and fumarase, as well as oxygen consumption rates in the presence of pyruvate, succinate, G3P, and quinone, were within the established control ranges (not shown).

**Table 2:** Enzymatic activities of the respiratory chain complexes I to IV (CI-CIV) and citrate synthase (CS) in permeabilized patient fibroblasts. Complex activities were measured by spectrophotometry and were normalized to citrate synthase activity (nmol.min-1.mg protein-1).

Complex/Enzyme.	Patient	Reference range
CI/CS	0.42	0.32–0.62
CII/CS	0.81	0.20–0.32
CIII/CS	2.9	1.4–2.5
CII + CIII/CS	0.64	0.29–0.55
CIV/CS	2.0	0.9–1.6
CS (*nmol.min*^*−1*^*.mg protein*^*−1*^*)*	29	31–65

## 4 Discussion

Here, we report functional characterization of the previously unreported c.890G > C *MIPEP* variant identified in a patient presenting with global developmental delay, infantile epileptic spasms syndrome, and hypotonia. We demonstrate that the substitution of glycine with alanine at position 297 impairs MIP-mediated processing in patient fibroblasts, resulting in a phenotype consistent with that previously reported, but without demonstrable defects in OXPHOS complex assembly or activity.

The initial series of four patients with *MIPEP* variants reported by Eldomery et al. presented with a severe phenotype consisting of developmental delay, hypotonia, seizures, elevated blood lactate and cardiomyopathy, predominantly non-compaction [6]. 2 out of 4 had dysmorphic features, and 3 out of 4 children died by 3 years of age. Pulman et al. expanded the clinical spectrum of COXPD31, reporting a patient with intellectual disability, hypotonia and cerebral atrophy, in whom the absence of cardiomyopathy was associated with improved survival at 20 years of age [12]. More recently, a patient reported by Wang et al. had hypertrophic cardiomyopathy, hypotonia, lactic acidemia and died in infancy supporting the association of cardiomyopathy with early death in COXPD31 [18]. The patient described herein presented in infancy with typical neurological features but only mild cardiac involvement, which has normalized over time, correlating with longer-term survival and clinical stability at 7 years of age.

The mechanism by which impaired MIP-mediated processing leads to mitochondrial disease is poorly understood. MIP and Oct1 substrates include proteins from all five OXPHOS complexes, including the inner membrane insertase OXA1L, which is critical for OXPHOS biogenesis [12], [15]. Cells lacking MIP/Oct1 may develop deficiencies in various respiratory chain complexes, but this is variable and may be minimal in some cases [6], [12]. Moreover, Wang et al. found reduced mitochondrial DNA copy number in blood from a patient with severe disease [18], suggesting a role of MIP in mtDNA maintenance consistent with studies in yeast [5]. However, mtDNA was elevated in fibroblasts from a patient with a milder phenotype and in hypomorphic cell lines, suggesting that the effect may be context-dependent, varying by tissue type or by the level of residual MIP activity. In the present case, we did not observe any significant changes in OXPHOS assembly or respiratory chain activity in fibroblasts, despite the presence of obvious defects in NDUFV2 and ATP5F1 processing. Thus, it appears that the c.890G > C *MIPEP* variant has a lesser impact on MIP activity and therefore on OXPHOS function, resulting in an intermediate phenotype.

Here, we observed that patient fibroblasts, despite a clearly impaired MIP-mediated substrate processing, do not exhibit OXPHOS deficiency in contrast to previous reports. A lack of biochemical or molecular defects in skin fibroblasts is not uncommon in mitochondrial disease [16]. For example, a homozygous missense variant in the mitochondrial phenylalanine tRNA synthetase, associated with early-onset epilepsy, caused CIV deficiency in skeletal muscle and myotubes, but not in skin fibroblasts [2]. Likewise, mtDNA deletions caused by *MPV17* variants were detectable only in skeletal muscle and were absent in patient fibroblasts [7]. In part, such findings reflect the glycolytic nature of fibroblast cultures and their low reliance on functional oxidative phosphorylation for survival. However, additional factors, such as tissue-specific gene expression, stability and the level of functional impairment of the mutant protein, and the presence of modifying factors, can cumulatively modify the sensitivity of different tissues to mitochondrial dysfunction, resulting in tissue-specific clinical presentations. For example, a study of fatal hepatopathy caused by *EFG1* variants nicely demonstrated that higher hepatocyte sensitivity may be the result of the interplay between residual levels of the mutant *EFG1*, low basal level of OXPHOS expression in liver, whereas compensatory mechanisms induced in other tissues, could contribute to their increased resistance to the effects of the mutation [3]. As a result, OXPHOS assembly in the liver was severely compromised compared to fibroblasts, muscle, and heart tissues in these patients. Analogously, the clinical presentation of different *MIPEP* variants may depend on residual MIP levels across tissues. However, because MIP acts upstream of OXPHOS assembly, the stability and functionality of the intermediate isoforms that accumulate in MIP-deficient tissues can be an important modifier, if not a determinant, of the severity of OXPHOS deficiency and disease presentation. Thus, we hypothesize that despite the decrease in MIP, its residual activity in fibroblasts is sufficient to maintain a pool of mature OXPHOS subunits, enabling the assembly and function of the OXPHOS complexes. However, in differentiated tissues, such as skeletal muscle, brain tissue or cardiomyocytes, MIP activity and the availability of fully processed OXPHOS subunits may become rate-limiting for efficient OXPHOS biogenesis. Our access to patient fibroblasts provides us with a practical way to test this hypothesis, either by directly converting them into cardiac or neuronal cells or by reprogramming them into iPSCs and differentiating them into disease-relevant cell types.

Compared to patient fibroblasts, a muscle biopsy is preferable for diagnosing mitochondrial disease. Because muscle fibers are highly metabolically active and depend on functional mitochondria for contractility, they are more likely to exhibit a biochemical or histological phenotype. In the case of *MIPEP*, Eldomery et al. reported that analyses of muscle biopsies from three patients revealed an accumulation of glycogen and the appearance of abnormal mitochondria, but respiratory chain (RC) activities in a less affected patient (P1) did not reveal a clear RC defect [6]. In the present case, a muscle biopsy was unavailable, which necessitated the use of patient fibroblasts. While this may have limited our ability to detect a clear OXPHOS deficiency, our clinical and genetic data, combined with the observed impaired processing of three known MIP substrates, and its restoration in complemented patient fibroblasts, support the deleterious nature of the identified variant.

## Funding and acknowledgement

This work was supported by the 10.13039/100013465French Muscular Dystrophy Association (AFM-Téléthon, #22529 and #29087) to MDM. BR acknowledges the support from the 10.13039/100013465AFM-Téléthon (#24318). LGS acknowledges funding from Cure Kids and the Health Research Council of New Zealand. EA was supported by a grant from The POLG Foundation to MDM. The authors thank the patient and his parents for their participation in the study.

## CRediT authorship contribution statement

**Benedetta Ruzzenente:** Writing – review & editing, Writing – original draft, Methodology, Investigation, Funding acquisition, Formal analysis, Conceptualization. **Pierre-Hadrien Becker:** Writing – review & editing, Methodology, Investigation. **Elissa Afram:** Writing – review & editing, Investigation. **Pauline Gaignard:** Writing – review & editing, Validation, Supervision, Resources, Methodology, Investigation, Formal analysis. **Agnes Rötig:** Writing – review & editing, Resources. **Denis M. Nyaga:** Formal analysis, Investigation, Writing – review & editing. **Cynthia Sharpe:** Writing – review & editing, Resources, Investigation, Formal analysis. **Lynette G. Sadleir:** Writing – review & editing, Resources, Funding acquisition, Formal analysis. **Bryony Ryder:** Writing – review & editing, Writing – original draft, Visualization, Validation, Supervision, Resources, Project administration, Methodology, Investigation, Formal analysis, Data curation, Conceptualization. **Metodi D. Metodiev:** Writing – review & editing, Writing – original draft, Validation, Supervision, Resources, Project administration, Methodology, Investigation, Funding acquisition, Formal analysis, Data curation, Conceptualization.

## Ethics statement

Informed consent was given before sample collection. This study adhered to the Declaration of Helsinki.

## Declaration of competing interest

LGS receives consulting fees from the Epilepsy Study Consortium. The authors have no conflict of interest to disclose.

## Data Availability

Data will be made available on request.
